# A versatile software package for inter-subject correlation based analyses of fMRI

**DOI:** 10.3389/fninf.2014.00002

**Published:** 2014-01-31

**Authors:** Jukka-Pekka Kauppi, Juha Pajula, Jussi Tohka

**Affiliations:** ^1^Department of Computer Science and HIIT, University of HelsinkiHelsinki, Finland; ^2^Brain Research Unit, O.V. Lounasmaa Laboratory, School of Science, Aalto UniversityEspoo, Finland; ^3^Department of Signal Processing, Tampere University of TechnologyTampere, Finland

**Keywords:** functional magnetic resonance imaging, naturalistic stimulus, re-sampling test, Matlab, grid-computing, GUI

## Abstract

In the inter-subject correlation (ISC) based analysis of the functional magnetic resonance imaging (fMRI) data, the extent of shared processing across subjects during the experiment is determined by calculating correlation coefficients between the fMRI time series of the subjects in the corresponding brain locations. This implies that ISC can be used to analyze fMRI data without explicitly modeling the stimulus and thus ISC is a potential method to analyze fMRI data acquired under complex naturalistic stimuli. Despite of the suitability of ISC based approach to analyze complex fMRI data, no generic software tools have been made available for this purpose, limiting a widespread use of ISC based analysis techniques among neuroimaging community. In this paper, we present a graphical user interface (GUI) based software package, ISC Toolbox, implemented in Matlab for computing various ISC based analyses. Many advanced computations such as comparison of ISCs between different stimuli, time window ISC, and inter-subject phase synchronization are supported by the toolbox. The analyses are coupled with re-sampling based statistical inference. The ISC based analyses are data and computation intensive and the ISC toolbox is equipped with mechanisms to execute the parallel computations in a cluster environment automatically and with an automatic detection of the cluster environment in use. Currently, SGE-based (Oracle Grid Engine, Son of a Grid Engine, or Open Grid Scheduler) and Slurm environments are supported. In this paper, we present a detailed account on the methods behind the ISC Toolbox, the implementation of the toolbox and demonstrate the possible use of the toolbox by summarizing selected example applications. We also report the computation time experiments both using a single desktop computer and two grid environments demonstrating that parallelization effectively reduces the computing time. The ISC Toolbox is available in https://code.google.com/p/isc-toolbox/

## 1. Introduction

Most neuroimaging studies, such as those based on functional magnetic resonance imaging (fMRI), have so far utilized relatively simple static stimuli to analyze brain functions (Spiers and Maguire, 2007). However, the human brain has evolved to function in a tremendously stimulating world and the investigation of complex brain functions, including socio-emotional or comprehension-related processes, is limited when using highly controlled/simplistic experimental setups, because these functions are only triggered under highly complex stimuli. There is an increasing interest in studying the human brain function with dynamic, continuous stimuli that are designed to be closer to normal everyday life than in conventional, strictly controlled research paradigms. The used stimuli can be, for example, a movie. This kind of fMRI data cannot be straight-forwardly analyzed based on a general linear model (GLM), because a GLM requires a reference time course of the task that is impossible to obtain for a multi-dimensional stimulus such as a movie, unless focusing the data-analysis on a specific feature of the stimuli. For this reason, new data-driven methodologies are needed. The use of novel experimental setups involving rich stimuli and data-driven analysis methods which are particularly designed to study complex brain functions opens up entire new fields for neuroscience research.

Inter-subject correlation (ISC) based analysis, originally introduced by Hasson et al. (2004), is a conceptually simple approach to analyze fMRI data acquired under naturalistic stimuli. In the ISC based analysis, the extent of shared processing across subjects during the experiment is determined by calculating correlation coefficient between the fMRI time series of the subjects in the corresponding brain locations. This way, ISC based analyses effectively avoid the modeling of the stimuli.

ISC based analyses have been previously applied to analyze fMRI data collected during complex stimuli or tasks, including movies (Hasson et al., 2004; Jääskeläinen et al., 2008; Kauppi et al., 2010b; Nummenmaa et al., 2012), TV news reports (Schmälzle et al., 2013), auditory and audiovisual narratives (Wilson et al., 2008), pieces of music (Abrams et al., 2013) and aesthetic performances (Jola et al., 2013). ISC based analysis has also been used for feature selection as a part of multivariate pattern analysis of data collected during a movie experiment (Kauppi et al., 2011). There can be different motivations to apply ISC based analysis for fMRI data. One can address specific neuroscientific research questions (some examples are provided in section 3) or simply try to make sense of highly complex fMRI data to generate new hypotheses. Whatever the motivation, it is important to keep in mind that the ISC is primarily a measure of *shared* hemodynamic activity across subjects and not a measure of hemodynamic activity *per se*. However, as shown by Pajula et al. (2012), when equipped with proper nonparametric statistical procedures (Kauppi et al., 2010b), ISC based methods can be used for detecting traditional fMRI activations without requiring specific, *a-priori* stimulus time course models.

Despite of the suitability of the ISC based approach to analyze complex fMRI data, no generic software tools have been made available for this purpose, limiting a widespread use of ISC based analysis techniques among neuroimaging community. Reliable and sophisticated ISC based analysis requires management of several nontrivial methodological, computational, and visualization related issues (such as heavy computational and memory load of the analysis, the choice of a proper ISC measure, handling non-standard statistical significance testing, and the visualization of multidimensional time-varying ISC maps). Hence, it is obvious that a toolbox solving these issues would be highly beneficial and can substantially simplify the use of the ISC based analysis among neuroscientists, consecutively advancing our understanding of complex human brain functions.

We have previously introduced a framework for the basic ISC based analysis (Kauppi et al., 2010b) and started building an open source, graphical user interface (GUI) based Matlab toolbox, termed the ISC toolbox, for a generic, ISC based analysis of fMRI. A set of visualization tools—particularly designed for the ISC analyses—are integrated to the GUI. In this paper, we describe the methods behind of the ISC toolbox that implements, in addition to the basic ISC analysis, many advanced ISC based computations such as phase ISC, time-windowed ISC, and comparison of ISCs between different stimuli. We will describe the analysis methods, explain the rationales behind them and demonstrate their potential use by reviewing selected example application studies.

As the ISC based analyses are data and computation intensive, the ISC toolbox is equipped with mechanisms to execute the parallel computations in a cluster environment automatically and with an automatic detection of the cluster environment in use. Currently, SGE-based environments [Unity Grid Engine (Univa Corporation, 2013), Son of a Grid Engine (Love, 2013), or Open Grid Scheduler (Scalable Logic, 2013)] and Slurm environment (Yoo et al., 2003) are supported. As there are ISC method-specific challenges in the parallelization, we will describe the automatic parallelization mechanisms in the paper. The ISC toolbox (the current version is 2.0) is available in https://code.google.com/p/isc-toolbox/

The organization of the paper is as follows. In section 2, after providing an overview of the toolbox, we will detail the ISC methods (section 2.2), describe the implementation of the toolbox (section 2.3), and briefly describe a set of visualization tools, customized to the ISC analyses (section 2.4). In section 3, we demonstrate the use of ISC-based analyses by reviewing selected studies. In section 4, as we consider cluster computing features of the toolbox important, we present the computation time experiments demonstrating the added value of parallel computing. Section 5 discusses current limitations and future directions of the toolbox and section 6 concludes the paper.

## 2. Materials and methods

### 2.1. Overview and usage of ISC toolbox

The ISC toolbox is designed for generic ISC based analysis of fMRI data. No information about the stimulus is required to carry out the analysis, making the toolbox suitable to analyze nearly any kind of fMRI data. Naturally, data from at least two subjects are needed for the analysis because the analysis procedure is based on voxel-wise correlations of fMRI time-series across subjects. A normal desktop computer equipped with the Matlab is sufficient to carry out the basic ISC analysis in many situations. However, in certain situations it is recommended to utilize a computer cluster to carry out the analysis. For instance, the use of cluster can be meaningful if the number of subjects is high (tens of subjects), advanced ISC analyses need to be computed, or reliable re-sampling based nonparametric statistical inference is needed to construct ISC maps. The toolbox can efficiently and automatically utilize cluster environment, allowing easy and fast ISC based analysis.

**Figure 1:**
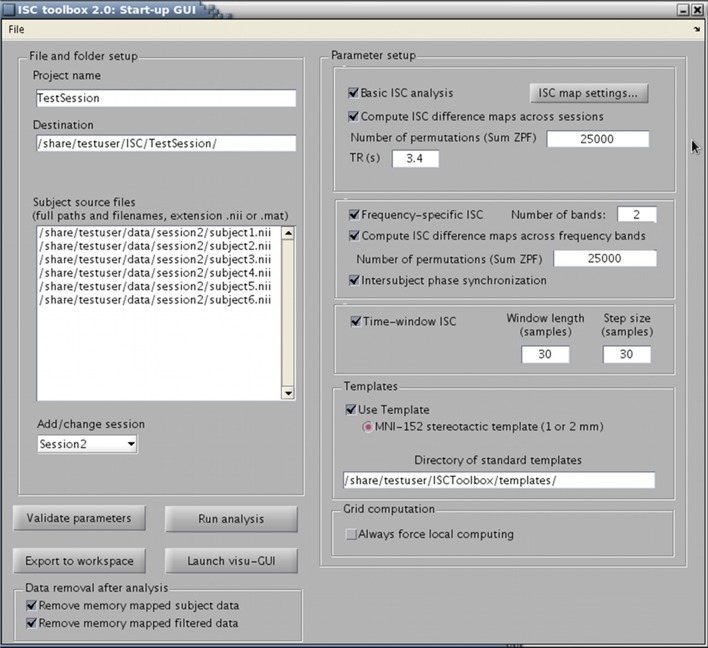
**ISC Toolbox's startup GUI where user can define the parameters for analysis, test them, run the ISC based computations and launch a separate GUI for visualization of the results**.

The toolbox consists of three parts: (1) a startup GUI for setting-up parameters for the analysis, (2) a main program that computes ISC maps based on selected parameters, and (3) a GUI-based visualization tool for the exploration of the findings. The GUIs are designed to make the analysis easier but a whole analysis pipeline can also be carried out from Matlab's command line. The main window of the startup GUI is shown in Figure 1 to demonstrate the main features of the ISC toolbox. Using the startup GUI, a user can easily select the appropriate analyses and their parameters. In the left side of the panel, a user chooses a descriptive project name and the destination folder of the analysis. For a large textbox (“Subject source files”), a user adds the names of the files containing fMRI time-series of the subjects used in the analysis. The toolbox assumes that fMRI signals have been preprocessed and preferably registered to a standard template. Preprocessing and registration algorithms are not implemented in the ISC toolbox because well developed free software packages exist for these purposes. Preprocessed and registered fMRI data sets of the subjects should be given either in nifti- or mat-format as 4-dimensional (a 3-dimensional position coordinate and time) matrices. If several acquisitions are available for each subject or acquisitions for more than one group are available, a user can analyze them all by adding more sessions to the project. The left side of the panel also contains buttons for parameter validation and for launching the main program which computes ISC maps once the parameters have been successfully validated. After running the main program, the visualization GUI to analyze results can be launched from the separate button. There is also an option to export parameters to Matlab's workspace (using the button “Export to workspace”). Automatic postprocessing operations can also be used to remove portions of data generated during the analysis to free disk space.

Different ISC analysis options are selected from the right side of the panel. A *basic ISC analysis* includes the generation of the ISC maps including thresholding of the maps based on a nonparametric statistical test. Details of this analysis can be specified from a separate panel under a button “ISC map settings.” If more than one session is added to the project, it is possible to compute *ISC difference maps* to investigate whether ISCs in some of the sessions (conditions) are higher than in the others. *Frequency-specific ISC* decomposes fMRI time-series of the subjects to frequency sub-bands and computes and thresholds ISC maps for each sub-band. *Time-window ISC* computes ISC maps for several consecutive time-frames. *Inter-subject phase synchronization* combines the localization of inter-subject similarities in space, time, and frequency. These analyses are explained in section 2.2.

An arbitrary volume size can be used to compute ISC maps as long as the volume is same across subjects. However, the GUI built for the visualization of the results assumes that all fMRI data sets have been registered to a common MNI152 template. The toolbox also assumes that Harvard-Oxford cortical and sub-cortical brain atlases are available to compute and visualize inter-subject similarities for selected brain regions. Hence, to allow convenient analysis of the results, it is highly recommended to register the data to the MNI template prior to ISC analysis as well as to have the Harvard-Oxford brain atlases available. The anatomical template, atlases, and the brain mask for limiting ISC computations only for the voxels within the brain are freely provided with the FSL software package. The directory including the corresponding nifti-files should be provided in the startup GUI (subpanel “Templates”). The use of a computational cluster can be disabled under the panel “Grid computation” if needed.

After all parameters have been set and validated, they are automatically saved under the project directory in a single structure array called “Params” (the parameters can also be saved or the existing parameters can be loaded by a user from the file-menu in the upper left corner). The main program performs all ISC based computations defined in this parameter structure array. The program saves intermediate and final results of the computations to the project folders. The visualization GUI allows flexible analysis of ISC maps over an anatomical template together with the brain atlases. It also allows exporting interesting data to Matlab's Workspace for customized analysis.

### 2.2. ISC methods

#### 2.2.1. Generation and visualization of the ISC maps

A correlation coefficient^1^ is a natural measure of similarity between fMRI time-courses of two subjects. ISC toolbox allows an analysis of the similarities in the time-courses across multiple subjects. We compute the mean of the voxel-wise correlation coefficients across all possible subject pairs as (Kauppi et al., 2010b):

(1) $$\bar{r}=\frac{1}{N(N-1)/2}\sum_{i=1}^{N}\sum_{j=2,j>i}^{N-1}r_{ij},$$

where *r* denotes a group-level ISC in a given voxel (a voxel index is omitted for clarity), *N* is the total number of subjects, and *r*_*ij*_ is the correlation coefficient between fMRI time-courses of subjects *i* and *j*. Note that because *r*_*ii*_ = 1 and *r*_*ij*_ = *r*_*ji*_, it is sufficient to compute correlation coefficients across *N*(*N* − 1)/2 subject pairs (instead of *N*^2^ pairs). However, because the number of subject pairs increases approximately quadratically with *N* and Equation (1) is computed for every voxel within the brain, it may be necessary to compute extremely high number of correlation coefficients (in the order of 10^8^) even for the most basic ISC analysis, rendering the analysis procedure computationally demanding.

We briefly explain our preference to *r* as the test-statistic, particularly over a related one used by Lerner et al. (2011). The main reason is that the test statistic *r* can be seen as an estimator of the true (but unknown) population ISC ρ under the model that ρ_*ij*_ = ρ + ϵ_*ij*_, where ρ_*ij*_ is the true correlation between subjects *i* and *j* and ϵ_*ij*_, with zero-expectation, models the between subject-pair variation. More specifically, if *r*_*ij*_ approaches ρ_*ij*_ and ρ_*ij*_ approaches ρ, then *r* approaches ρ. Lerner et al. (2011) computed the average correlation of the subject time course and average time course of remaining subjects. This is closely related to *r* statistic^2^ and neither one seems to be quantitatively better than the other. However, the statistic in Lerner et al. (2011) cannot be straight-forwardly interpreted as an estimator of the population ISC in an above sense, which results in our preference of *r*.

#### 2.2.2. Nonparametric re-sampling test

The correlation coefficients *r*_*ij*_ in Equation (1) are not independent because each subject is present in more than one subject pair (e.g., *r*_*ij*_ and *r*_*kj*_ are overlapping because they both depend on the same time-series measured from subject *j*). Also, it is well known that BOLD-fMRI signals are temporally correlated. Therefore, the standard tests for assessing the significance of *r* are not valid. We use a fully nonparametric re-sampling based method to evaluate the significance of *r* (Kauppi et al., 2010b). In this method, we perform a test against a null hypothesis that *r* statistic is the same as for data with no specific time-structure. To compute a “null” re-sampling distribution, we circularly shift each subjects time-series by a random amount so that they are no longer aligned in time across the subjects, and then calculate *r* statistic. This way we can account for temporal autocorrelations present in the fMRI data. In practice, calculation of all the possible time shift combinations is computationally prohibitive and the distribution is approximated with finite number of realizations, randomizing the experiment across voxels and time-points, by default 100 million realizations are generated. To obtain critical thresholds for significant ISCs, we first compute *p*-values of the true realizations for each voxel based on the null distribution and then correct the values using the false discovery rate (FDR) based multiple comparisons correction (Benjamini and Hochberg, 1995). Using our visualization tool, it is possible to investigate thresholded ISC maps over an anatomical template with different critical thresholds.

#### 2.2.3. Parametric t-test

The ISC toolbox contains an option to threshold group-level ISC maps also based on a simple parametric test proposed by Wilson et al. (2008). For this test, correlation coefficients are first transformed to z-scores using a Fisher's z transformation:

(2) $$z_{ij}=\frac{1}{2}\log\left(\frac{1+r_{ij}}{1-r_{ij}}\right).$$

Then, a one-sample *t*-test with *N*(*N* − 1)/2 − 1 degrees of freedom is performed under a null hypothesis that the ISC is zero. Note that the independence assumption of the observations made by the test is violated in practice.

#### 2.2.4. Generation and visualization of the ISC difference maps

With the ISC toolbox, it is also possible to generate and visualize ISC *difference maps* to investigate if there are significant differences in the ISCs between two conditions. For instance, in studies where same subjects are scanned twice under different stimuli, it can be highly interesting to analyze whether or not ISC was stronger in one of the conditions. We use a modified Pearson-Filon statistic based on Fisher's z-transformation (ZPF; Raghunathan et al., 1996) for this purpose, which is a recommended statistic for testing if two nonoverlapping but dependent correlation coefficients are different (Krishnamoorthy and Xia, 2007). Consider four time-series **t**^*a*^_*i*_, **t**^*a*^_*j*_, **t**^*b*^_*i*_ and **t**^*b*^_*j*_ measured from two subjects *i* and *j* in two conditions *a* and *b*. The corresponding correlation coefficients *r*^*a*^_*ij*_ and *r*^*b*^_*ij*_ are nonoverlapping, because they have been computed using different time-series. However, stimuli used in two conditions *a* and *b* may not be independent, making a dependency assumption plausible. We extend the pairwise ZPF statistic for group-level analysis by combining the pairwise statistic from all subject pairs, and design a fully nonparametric test to assess the significance of the resulting group-level statistic (Reason et al., under review). Our final “sum ZPF” statistic is given by:

(3) $${\text{ZPF}}_{\Sigma ij}^{ab}=\sum_{i=1}^{N}\sum_{j=2,\;j>i}^{N-1}\frac{(z_{ij}^{a}-z_{ij}^{b})\sqrt{(T-3)/2}}{\sqrt{1-\text{cov}(r_{ij}^{a},r_{ij}^{b})}/\left[\left(1-{\left(r_{ij}^{a}\right)}^{2})(1-{\left(r_{ij}^{b}\right)}^{2}\right)\right]},$$

where *z*^*a*^_*ij*_, *z*^*b*^_*ij*_ are the Fisher's z transforms [see Equation (2)] of the correlation coefficients *r*^*a*^_*ij*_, *r*^*b*^_*ij*_, respectively, *T* is the length of a time-course and cov(*r*^*a*^_*ij*_, *r*^*b*^_*ij*_) is a large scale covariance (Raghunathan et al., 1996). The test is performed under the null hypothesis that each ZPF value is drawn from a distribution with zero mean, which occurs when there is no difference in ISC between the conditions. The approximate permutation distribution is generated by randomly flipping the sign of pairwise ZPF statistics before calculating Equation (3) using a subsample of all possible random labelings. Maximal and minimal statistics over the entire image corresponding to each labeling are saved to account for multiple comparisons by controlling family-wise error rate (FWER; Nichols and Holmes, 2002). Due to the symmetry of the distribution, thresholds for both directions are obtained with this procedure. The default number of random permutations over the whole image is 25,000.

**Figure 2:**
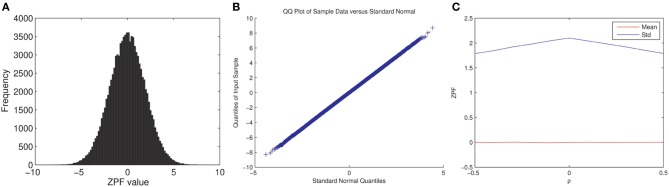
**ZPF Monte Carlo simulation**. We simulated fMRI time series for two subjects and two conditions as **x**^*a*^_*s*_ = β**t**^*a*^ + **n**^*a*^_*s*_, where **x**^*a*^_*s*_ (*s, a* = 1, 2) are the simulated time series, **t**^*a*^ is the common time-series between the two subjects in the condition *a*, **n**^*a*^_*s*_ is pink noise [generated as in Pajula et al. (2012)], and β is selected so that the true correlation between the subjects' time-series is ρ, which is varied during the simulation. **t**^*a*^ was generated by smoothing white Gaussian noise with a box filter and convolving the resulting time series by a hemodynamic response function. The times series length was 100 with modeled TR of 2*s*. Note that ρ has the same value for both conditions, according to the null-hypothesis. The simulation does not model for the dependence between the two conditions. The simulation was repeated 100000 times for each ρ = −0.5, −0.4, …, 0.5. **(A)** Shows the histogram of ZPF values when ρ = 0.3, **(B)** Shows the QQ-plot for the same case, and **(C)** Shows the average value and the standard deviation of the ZPF-statistics as a function of ρ, where a slight dependence of the standard deviation on ρ is observed. As it is visible (1) the distribution of the ZPF was Gaussian (with a larger variance than 1), and (2) its parameters did not markedly depend on the value of ρ.

Note that we cannot readily confirm the full exchangeability under the null hypothesis for the permutation test since: (1) fMRI time series are autocorrelated and (2) the subject pairs are not independent. Assuming temporal independence and normality, the ZPF-statistic can be shown to be distributed according to the standard normal distribution under the null hypothesis of no correlation difference (Raghunathan et al., 1996), which is enough to ensure the correctness of the test (Good, 2005). However, it is unclear to what extent this distributional result holds for the ISC analysis. We performed here a simple Monte Carlo simulation that verified that the ZPF statistics are normally distributed with a constant variance, not dependent on the (true) values of *r*^*a*^_*ij*_ = *r*^*b*^_*ij*_, thus partially verifying the permutation test. The experiment and its results are summarized in Figure 2.

#### 2.2.5. Frequency-specific ISC analysis

The ISC toolbox contains an option to analyze ISCs in distinct frequency sub-bands. The approach is well-motivated because real-world events and stimuli unfold over multiple time-scales (Kauppi et al., 2010b). For instance, features of visual stimuli, spoken sentences, or the development of social interaction may unfold over very different time-scales. Thus, it is plausible to assume that the brain processes information in distinct frequency sub-bands. In the frequency-specific ISC analysis, we first filter the original time-series of each voxel (and subject) to multiple frequency sub-bands using an octave filter bank based on stationary wavelet transformation (SWT; Kauppi et al., 2010b). After band-pass filtering each fMRI time-series, we compute ISCs using the Equation (1) voxel-wise separately within each frequency sub-band and threshold the ISC maps using the same test as described in section 2.2.1.

It has been shown previously that wavelets are well-suited to analyze fMRI data because of certain properties of the cortical fMRI time-series, such as 1/f -like frequency characteristics (Bullmore et al., 2004). Moreover, the SWT algorithm is specifically suited to our analysis because it performs a time-invariant (Bradley, 2003) transformation unlike the discrete wavelet transform (DWT). In practice, this property means that a small difference in the hemodynamic delays of two fMRI time series transforms into a similar small difference in the filtered signals, allowing consistent estimation of the correlation coefficients between the subjects time-series after performing the filtering. For the DWT, even a minor delay between two identical input signals might cause a large difference in the filtered signals, making it much less-suited algorithm for frequency-specific ISC analysis. The SWT algorithm can be efficiently implemented using a subband coding scheme based on successive decimations of so called quadrature mirror filters (QMFs) and convolution operations (Vetterli and Kovačević, 1995).

We use Daubechies scaling and wavelet functions as a default filter option as they satisfy a necessary QMF relationship (Vetterli and Kovačević, 1995) and have been successfully applied to fMRI data earlier (Bullmore et al., 2001; Achard et al., 2006). The maximum degree of the polynomials the scaling function can reproduce is called the number of the vanishing moments. The number of Daubechies filter coefficients are associated with the number of the vanishing moments by the equation *K* = 2*V*, where *K* is the number of filter coefficients and *V* is the number of vanishing moments. We use short filters of length *K* = 4 as a default analysis option which are flexible enough to encode polynomials with two coefficients (both constant and linear signal components). In principle, the localization in the frequency domain could be improved by using higher filter lengths, but the use of long filters increases computation time (SWT needs to be computed separately for the time-series of every subject for each brain voxel) and makes the detection of rapid signal changes less accurate. In addition, because typical fMRI measurements contain relatively low number of time points, short filters are preferred to minimize boundary artifacts. Daubechies basis functions are optimal in the sense that they provide the shortest filter length for the given number of vanishing moments. However, also other basis functions have been proposed for fMRI data analysis. For instance, Ruttimann et al. (1998) used symmetric spline wavelets because of their phase-preserving property.

#### 2.2.6. Time window ISC analysis

When analyzing complex fMRI data sets such as those collected during a movie watching, it is likely that ISCs vary drastically over the experiment. To analyze how ISC varies over time, it can be highly useful to compute ISC maps for several consecutive possible overlapping time windows. With the ISC toolbox, a user can specify suitable time window parameters (window length, step length between two consecutive windows) and compute “short-time ISC maps” for each window. To obtain these maps, we compute *r* statistic (across all voxels and subjects) within each time-window and assess the significance of the ISCs as described above. We randomize the generation of the null distribution across all time windows which leads to a common threshold for all windows. The length of the time window has to be sufficient to obtain reliable estimates of *r* for each time window. The choice depends on the number of subjects and the type of the stimulus. Therefore, it is not straight-forward to give exact suggestions about the minimal time-window length. However, window lengths as short as 10 samples have been used (Nummenmaa et al., 2012).

The toolbox allows the visualization of the time window ISC maps over an anatomical template. It also automatically computes the mean of *r*-values across voxels within different brain region-of-interest (ROIs), allowing plotting ROI-averaged ISCs over time. These curves can be correlated with the features of the stimuli, behavioral ratings or other variables of interest.

#### 2.2.7. Intersubject phase synchronization

Time window ISC and frequency-specific ISC analyses can provide neuroscientifically meaningful insights into complex fMRI data. An obvious way to combine benefits of both approaches is to compute frequency-specific ISC maps in several time windows to investigate temporal evolution of the ISCs in specific time-scales. ISC toolbox automatically computes also these maps if the user performs both time window ISC and frequency-specific ISC analyses. A limitation of this approach is that the temporal resolution of the analysis can be modest because each time window must contain several time points to allow meaningful interpretation of the correlation coefficient. This problem is most prominent in the lowest frequency sub-bands because the temporal resolution of slow fluctuations is inherently poor as stated by the time-frequency uncertainty principle (Cohen, 1995). To increase the temporal resolution of the time-varying analysis in distinct frequency sub-bands, we propose using phase synchronization between subjects as a measure of inter-subject similarity. A similarity measure based on instantaneous phase allows the analysis of the band-pass filtered signals on the basis of inherent temporal resolution of the time series. This is in contrast to the time window ISC analysis for which the resolution is further limited by the length of the time-window.

Many phase synchronization measures have been designed to analyze functional neuroimaging signals (Vinck et al., 2011) but they are mainly used to analyze electroencephalography and magnetoencephalography signals. Unlike these signals, fMRI time-series may not be characterized by oscillatory activity. However, the analysis of the instantaneous phases still remains a valid method to characterize a specific interrelation between phases (Pikovsky et al., 2000; Laird et al., 2002). To extract phase information, complex-valued analytic time-series must be available. Hence, we apply the Hilbert transform (Goswami and Hoefel, 2004) to the fMRI time-series to obtain their corresponding analytic signals^3^. We take the *absolute angular distance* (Vinck et al., 2010) between the time-series of two subjects as a dissimilarity measure:

(4) $$p_{ij}(t)=|\theta_{i}(t)-\theta_{j}(t)|\;\mathrm{mod}\;\pi ,$$

where *t* is a time-point index and angles θ_*i*_, θ_*j*_ are computed based on the analytical time-series measured from subjects *i* and *j*. This is an intuitive measure of phase interrelationship between the fMRI time-series of two subjects: If fluctuations of the (band-limited) time-series between subjects are highly similar, it is expected that the absolute phase difference is smaller than when fluctuations are different. There are different possibilities to extend this measure to group-level analysis (Glerean et al., 2012). We use a comparable definition to our ISC measure [Equation (1)] and compute the average of all subject-pairwise absolute angular distances as:

(5) $$\bar{p}(t)=\frac{1}{N(N-1)/2}\sum_{i=1}^{N}\sum_{j=2,\;j>i}^{N-1}p_{ij}(t).$$

Our final measure of inter-subject phase synchronization (IPS) is the normalized version of *p*:

(6) $$\hat{\bar{p}}(t)=1-\frac{\bar{p}(t)}{\pi }.$$

This measure has its values always within the range [0 1], where the value 1 indicates a complete phase similarity and the value 0 corresponds to a complete *absence* of phase similarity across subjects. Similarly to time window ISCs, the ISC Toolbox allows different plotting options for IPS results. For instance, averaged IPS values within selected ROIs can be plotted over time. These curves can be then correlated with the features of the stimuli or other variables of interest.

### 2.3. Implementation

As explained in section 2.1, the use of the ISC Toolbox starts from the startup GUI where a user defines requested analyses and their parameters (see Figure 1). The GUI automatically detects the operating system and checks that all necessary software and files are available. After a user has selected desired analysis options, the GUI validates them. After a successful validation, the parameters are set in a structure array called *Params* which is saved in a mat-file. The GUI also generates the destination directory and all necessary sub-directories for the analysis results.

The computational analysis is controlled inside the main function named *runAnalysis*. The Matlab code of this function is grouped in six computational stages to clarify how the computations can be distributed across a computer cluster:

Stage 1 Binary data files for the analysis results as well as the memory map pointers to access these files are initialized. The pointers are saved in the structure called *memMaps* which is saved in the analysis destination directory. In the later stages of the program, the files are repeatedly accessed and modified using these pointers (see more information about the Matlab's memory mapping feature below).
Stage 2 The wavelet filtering for the frequency-specific ISC analysis is performed.
Stage 3 Average ISC maps are computed, including the generation of the re-sampling distributions for the assessment of statistical thresholds.
Stage 4 Critical thresholds are calculated based on the re-sampling distributions including threshold correction for multiple comparisons. In the FDR-based correction, *p*-values for statistically significant (before a multiple comparison correction) samples need to be available. These are estimated in a nonparametric fashion from the observations of the re-sampling distribution using a linear interpolation.
Stage 5 Inter-subject synchronization curves over time are computed for the time window ISC and IPS for all the brain regions and thresholds defined in the Harvard-Oxford sub-cortical and cortical atlases.
Stage 6 All the generated statistical maps in the previous stages are saved to the analysis destination folder as nifti files. This stage is always computed locally even if a grid enviroment would be available.

The grouping of the code is based on the dependencies of the analysis pipeline: the execution of the functions within any of the stages is always dependent on the results of the preceding stage and therefore cannot be performed before all previous stages have been completed and their intermediate results have been saved to the analysis destination directories. However, computations inside the loop structures *within* each computational stage are independent of each other, meaning that functions repeatedly called inside these loops can be equally well run in parallel. In practice, a user does not need to understand how the code is written because the program can automatically parallelize computations across a computer grid/cluster.

Only those stages corresponding to ISC based analyses that are requested by the user are run when executing *runAnalysis*. For example, if the frequency-specific ISC analysis is not chosen by a user, the stage 2 is skipped.

Matlab's memory mapping is a mechanism that maps a portion of a file, or an entire file, on disk to a range of addresses within an application's address space. The application can then access files on disk in the same way it accesses dynamic memory (The Mathworks Inc., 2013). This memory mapping mechanism is employed in the ISC Toolbox for three main reasons:

1. Because of a large memory demand, all the data cannot be held in the central memory all the time.
2. The traditional file I/O can be very slow especially in cluster computing environments.
3. The memory mapping provides a mechanism for sharing the memory between multiple processes that is important for the cluster computing abilities in the ISC Toolbox.

The disadvantage of the used memory mapping mechanism is that it is highly hardware and also somewhat operating system and Matlab version dependent. The memory mapped data can become corrupt or unreadable if the used hardware or the Matlab version is changed. In the ISC Toolbox, the problem is circumvented by saving the important results out from the memory maps to nifti files. The corrected statistical thresholds are saved as Matlab's mat-file and also as a text file. Therefore, the visualization of the thresholded maps can be done afterwards easily with any visualization software. The memory mapping has been previously used in the SurfStat software within brain imaging (Worsley, 2008).

A heavy computational burden is one of the major issues when using the ISC Toolbox. Computations require large memory as mentioned already and they also take a long time to compute. Currently, the ISC Toolbox supports cluster computing in SGE-based (Oracle Grid Engine, Son of a Grid Engine, or Open Grid Scheduler) and Slurm (Simple Linux Utility for Resource Management) environments. Generally, the SGE based parallelization (Love, 2013; Scalable Logic, 2013; Univa Corporation, 2013) has been used extensively within brain imaging software such as FSL. The Slurm grid engine (GE) (Yoo et al., 2003) is currently becoming more common and for this reason also Slurm based parallelization was selected to be supported in the ISC Toolbox. The only requirements to use parallelization procedures in the toolbox are that the operating system of the used computer must be Linux and the user must have access to system running on one of these two GEs.

In both cases (SGE or Slurm), separate shell scripts must be generated for each computational stage before distributing them to the GE. The script generation and submission to GE is handled with the function *gridParser*. The *gridParser* function generates separate shell scripts for each process stage of each possible parallel process and submits these to the current GE. Simplified examples from the shell scripts generated by *gridParser* for the first stage of execution are presented in Listings 2.3 and 2.3. In this example, the project name is “ISC_test_analysis,” which defines the mat-file name for the Params struct. *memMapData* function implements the stage 1 of the analysis. The only input for the function is the Params struct. The number of generated scripts varies from 4, for the basic analysis using a single CPU, to hundreds depending on the selected analyses and the degree of parallelization.

**Listing 1:**
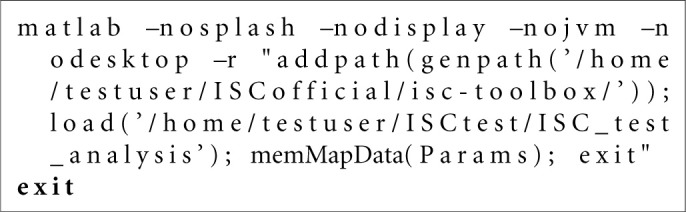
Bash script example for the Stage 1 of the analysis generated by the *gridParser* function for the SGE environment.

**Listing 2:**
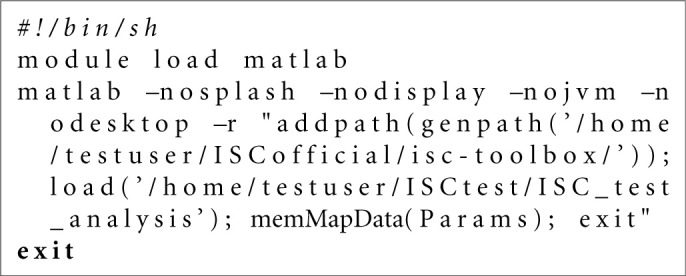
Bash script example for the Stage 1 of the analysis generated by the *gridParser* function for the Slurm environment.

The monitoring of the submitted tasks is handled with the function *waitGrid*. The function requests the running processes in the GE in defined time interval and prevents the main function to continue before all submitted sub-processes are finished.

The data integrity is always a critical question within parallel computing. It must be ensured that any two processes are not interfering each other and all data are saved safely. The function *freeToWrite* was developed to maintain the data integrity. It handles a specific lock system to ensure that only one process updates the memory maps at once. The lock is based on a simple lock-file which is generated before the data are going to be saved and deleted when the saving process has been finished. Every process which updates the memory maps are using the lock system. To simplify the debugging, every lock file has its own identifier based on the name of the process and the current process ID from the GE.

### 2.4. Visualization GUI

**Figure 3:**
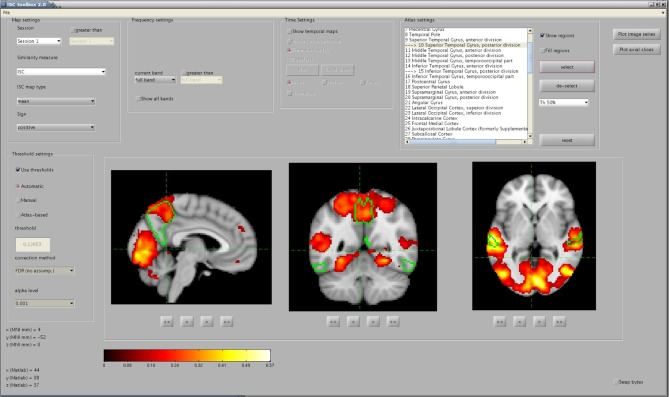
**The main window of the visualization GUI**. In the shown analysis example, a user has located significant ISCs in several brain areas, including the precuneus cortex and the posterior division of the superior temporal gyrus, whose perimetries are shown in green color over the anatomical template. The map was thresholded and FDR corrected (*q* < 0.001) over the whole brain using the re-sampling test of section 2.2.1.

To simplify the investigation of the ISC analysis results a separate visualization GUI, shown in Figure 3, was developed to interactively show the statistics maps and other results computed by the ISC Toolbox. The visualization GUI can show all the statistical maps resulting from the analyses by the toolbox. There are several software tools for high quality, interactive visualizations of the statistical maps from neuroimaging analyses. However, as far as we know, none of these is suitable for the visualization of advanced ISC analysis such as 4-D statistical maps of the time window ISC. In addition, a specialized visualization application provides additional convenience by allowing user to switch between different analysis results by a quick button press instead of a cumbersome reloading of the statistical maps one-by-one from a disk. We avoid the re-loading of the statistical maps by directly accessing the data portion of interest from the disk. The fast random access to the data is possible because the ISC results were mapped to a disk during the main analysis procedure with the aid of memory-mapping. Most of the data which are presented or used for creating the visualizations in the GUI are precomputed by the main analysis procedure and mapped to a memory. The memory-mapping minimizes the need of RAM, which enables the efficient interactive visualization and exploration of the analysis results also with slower computers. A price to pay for this added flexibility are possible cross-platform incompatibility issues, mentioned already in section 2.3, if the actual analysis is carried out with a different hardware than with which the analysis results are viewed. The simplest of these issues is the endianness, which can be changed by ticking the checkbox “Swap bytes.”

In addition to minimizing the memory consumption and the access time to the data in a disk, it is important to minimize the time that is spent for plotting accessed data on the screen. In Matlab, the plotting of the images is much faster when using indexed images (in an integer format) than using true color images or intensity images in a floating point format. Indexed images are fast to visualize because they use direct mapping of pixel values to colormap values. Hence, to maximize the browsing speed, the GUI converts ISC maps and anatomical templates from a floating point format to an integer format and combines these data into a single matrix of integer values. An appropriate colormap is then created to allow visualization of the indexed image on the screen in multiple colors. The colormap involves hot (yellow and red), cold (magenta and blue), and gray colors which allows the visualization of positive and negative ISCs as well as anatomical intensity values over a single image.

The exact appearance of the Visualization GUI on the screen depends which analyses, described in section 2.2, the user has run. For example, if only the basic ISC analysis has been run the visualization GUI enables only the analysis of ISCs across a whole session and frequency-spectrum by disabling “temporal settings” and “frequency settings” -panels. Statistical maps are shown in sagittal, coronal and axial views. The MNI coordinates of the views can be changed via the buttons below the axis. An additional option is to visualize several axial slices across the whole brain volume in a single figure. The exploration of the volume along a fourth dimension (time interval or frequency range) currently requires a button press. A user can also select Harvard-Oxford probabilistic atlas regions for the visualization over the statistical map and it is possible to view average ISCs for selected ROIs as a function of time. In addition to these visualizations, the GUI contains more advanced visualization options which allow detailed localization of ISCs in spatial, temporal and spectral dimensions.

The GUI allows fast and comprehensive visualization of the ISC analysis results in an exploratory manner. However, to address specific research questions, a further analyses not supported by the ISC Toolbox may be needed. Moreover, it may also be meaningful to customize the way how the results are visualized. For these purposes, the GUI has an option to export ISC maps and other results to the Matlab's workspace as variables. Although the ISC analysis results are also saved in a disk as Nifti-files and are freely accessible for a user, the export option allows quick and easy visualization of the threholded maps over an anatomical image and selected atlas regions for the dimensions (spatial, temporal, and spectral) of interest.

## 3. Applications

Next, we shortly exemplify how the toolbox has been successfully used to analyze fMRI data.

### 3.1. Basic ISC analysis for activation detection

A primary interest in many fMRI based imaging studies is to detect brain locations associated with a task related neural activity. Traditionally, this is achieved by a GLM based analysis, where voxel time courses are compared to the task-derived reference time course. The application of the GLM requires explicit knowledge how stimuli are varied during the experiment and cannot therefore be used to detect activations from experiments involving complex naturalistic stimuli. Pajula et al. (2012) showed that our “basic” ISC analysis described in section 2.2.1 is a suitable method to detect task related neural activation without making any assumptions about the applied stimuli. In this study, fMRI data from 37 right-handed subjects who all had performed the same five blocked design tasks^4^ were analyzed with both ISC Toolbox and a GLM based method. The idea is that the GLM-detected activations with this kind of strictly controlled and well-known tasks can be assumed to be reliable and can be treated as a gold standard. Interestingly, the comparison of the statistical maps of ISC and GLM revealed high agreement of the findings. This demonstrates that the ISC analysis can detect truly active brain regions in a manner that is completely “blind" to stimuli, making it highly promising method for detecting activity in data sets collected under naturalistic stimuli experiments.

### 3.2. ISC difference maps for analysis of aesthetic experiences

Understanding how spectators' brains process information during an aesthetic performance, such as a dance performance, is an interesting topic in neuroscience. To investigate this, videos of aesthetic performances can be shown to subjects while their brain activity is being measured using the fMRI. Stimuli in these experiments are very rich, making ISC based methods a natural choice for data analysis. Reason et al. (under review) used ISC toolbox to study whether auditory stimulation have an effect on the kinesthetic experience and/or the aesthetic appreciation of the spectator while watching dance. In the study, fMRI signals were acquired from 22 subjects under two different stimulus conditions: (1) a full audiovisual dance performance accompanied by the soundscapes of Bach (condition = “Bach”), and (2) the same dance performance without the music, including only visual stimuli as well as sounds of breathing and footfalls of the dancer (condition = “Breathing”). ISC toolbox was used to construct individual ISC maps of both conditions as described in section 2.2.1 as well as to construct ISC difference maps “Bach”<“Breathing” and “Bach”>“Breathing” as described in section 2.2.4.

The individual ISC maps showed large overlap in the visual and auditory cortices for both conditions. However, the analysis of the ISC difference maps revealed clusters in the temporal cortex that were unique to the different audio conditions, indicating also clear differences between the processing of the sound in the “Bach” and “Breathing” conditions. Based on detailed investigation of the ISC difference maps, Reason et al. (under review) suggested several possibilities how the presence or absence of music may influence spectators' experience. For instance, the postcentral gyrus of parietal cortex (BA 7) showed significantly greater ISC in the “Breathing” condition. The area is known for simultaneously processing multiple sensory modalities, in particular the somesthetic modality that includes touch. This somesthetic connection implies a form of motor cognition and could suggest that the “Breathing” elicited greater engagement of action understanding within body-specific mechanisms.

### 3.3. Frequency-specific ISC for analysis of temporal brain hierarchy

In our previous study (Kauppi et al., 2010b), we performed frequency-specific ISC analysis to investigate processing of movie events that occur over multiple time-scales. We analyzed fMRI data collected from the experiment (Jääskeläinen et al., 2008) where 12 subjects watched the 36 min clip of an Academy Award winning drama movie Crash (Lions Gate Films, 2005, directed by Paul Haggis; the movie was presented with sound). We constructed both frequency-specific ISC maps described in section 2.2.5 as well as ISC difference maps to compare differences in ISCs between distinct frequency subbands (see section 2.2.4).

The frequency-specific ISC analysis provided novel and interesting insights into the highly complex fMRI data. For instance, the analysis revealed that visual cortical ISC was present across the whole frequency spectrum of the fMRI signal, ISC in temporal areas occurred in all but the highest frequency band, and frontal cortical ISC was present only in the two lowest frequency bands. Hence, the frequency range showing significant ISC *contracted* when moving from lower-order sensory areas toward higher-order cortical areas. There are several possible explanations for the mappings found in this study. For instance, the findings might reflect the hierarchy of temporal receptive windows (TRWs) in the human brain, with sensory visual cortical areas showing short TRWs, and the TRWs becoming progressively longer as one ascends to functionally higher-order cortical areas (Hasson et al., 2008).

### 3.4. Time window ISC for analysis of higher-order brain functions

The use of movies as stimuli in neuroimaging studies offers new possibilities to understand higher-order brain functions, such as those related to social cognition and emotions. Nummenmaa et al. (2012) used the time window ISC (which they call moment-to-moment ISC) to analyze how ISC is associated with events that elicit emotions in movies. Functional MRI data from 16 subjects were collected while they watched movies depicting unpleasant, neutral, and pleasant emotions. After scanning, participants watched the movies again and continuously rated their experience of pleasantness–unpleasantness (i.e., valence) and of arousal–calmness. Short-time ISCs for each voxel were then computed using the ISC toolbox as described in section 2.2.6, using a 17-s sliding window (a step size of the time-window was one time point). Time series of valence and arousal ratings were then used to predict temporal variation of ISCs within each voxel.

Negative valence was associated with increased ISC in the emotion-processing network (thalamus, ventral striatum, insula) and in the default-mode network (precuneus, temporoparietal junction, medial prefrontal cortex, posterior superior temporal sulcus). High arousal was associated with increased ISC in the somatosensory cortices and visual and dorsal attention networks comprising the visual cortex, bilateral intraparietal sulci, and frontal eye fields. It was proposed that negative valence synchronizes individuals brain areas supporting emotional sensations and understanding of anothers actions, whereas high arousal directs individuals attention to similar features of the environment.

### 3.5. IPS for time-varying analysis of naturalistic fMRI data

IPS described in section 2.2.7 is an alternative option for time window ISC to analyze complex fMRI data over time. Glerean et al. (2012) applied both time window ISC and IPS analysis for naturalistic fMRI data collected from 12 subjects while they watched a feature movie (for details of the experiment, see Lahnakoski et al., 2012). IPS was computed within a frequency-band of 0.04–0.07 Hz and a time window ISC was computed for several window sizes from 4 to 32 samples (corresponding to window lengths from 8 to 64-s with the TR of 2-s) using a sliding window.

A major conclusion of the study was that the IPS approach provided improved temporal resolution as compared with the time window ISC. In addition, an anatomical mapping of the whole-brain temporal average of the IPS was highly consistent with the anatomical mapping of the ISC computed across the whole movie experiment (without using time windows), indicating that the IPS is a realiable measure of inter-subject similarity.

## 4. Computation time

The computation time was measured in three different hardware setups utilizing the both the local and distributed computing abilities of the ISC Toolbox. The local computations were tested with Dell Optiplex 755 desktop computer equipped with Intel Core2Duo E8400 CPU @ 3.00 GHz and 5GB read access memory (RAM). The distributed computations were tested in two computing clusters. The larger cluster, called Merope, had nodes running on HP ProLiant SL390s G7 equipped with Intel Xeon X5650 CPU 2,67 GHz and minimum of 4 GB RAM / core. The GE was Slurm. The smaller of the tested computing clusters, called Outolintu, was running with SGE and had nodes running on IBM System x3550 equipped with two Intel Xeon X5450 CPUs 3.0 GHz and 32 GB RAM (with 10 GB swap) for each node.

In the Merope cluster, on average 32 processes were run simultaneously. With the Outolintu cluster the maximum of parallel processes was limited to 10 due to global usage limitations for a single user of this cluster. The computing times of cluster environments were averaged from three separated runs as in the cluster the computing time can be affected from the current load of the cluster as well as the implementation of the distributing system causes a small variation on computing time.

The computing time was measured from “the user perspective”: Starting from the moment when user pushes the “Run Analysis” -button of the startup GUI to the moment when the analysis was finished. In a cluster environment, this means that the processing times of the GE were included to the total processing time.

The analyses were performed for the same measurement data which was used in earlier studies with the ISC Toolbox (Jääskeläinen et al., 2008; Kauppi et al., 2010b). The data was acquired from 12 subjects (*TR* = 3.4 s, 244 time points) and was registered to MNI152 space (for details see Kauppi et al., 2010b). The image dimensions were 91 × 109 × 91 × 244 (X × Y × Z × time) which resulted in an 840 MB file size for each subject and 9.8 GB total size of the analysis data set.

The tested ISC Toolbox setups were “basic ISC”, “basic ISC + time window ISC,” and “basic ISC + frequency-specific ISC.” The first setup computed the ISC map across the entire length of the time-series and constructed a re-sampling distribution based on 100 million random shufflings of the time-series as in our earlier study (Kauppi et al., 2010b). The second setup was similar to the first setup except that the time window ISC with the window length and window step of 30 samples was used in addition to the basic ISC analysis. The third setup used frequency-specific ISC with three frequency sub-bands instead of time window ISC. The number of randomizations to threshold the ISC *r*-maps was the same as in setups 1 and 2, and 25,000 random permutations for each brain voxel was used to construct a null permutation distribution of the sum ZPF statistic to allow thresholding of ISC difference maps between frequency bands.

**Figure 4:**
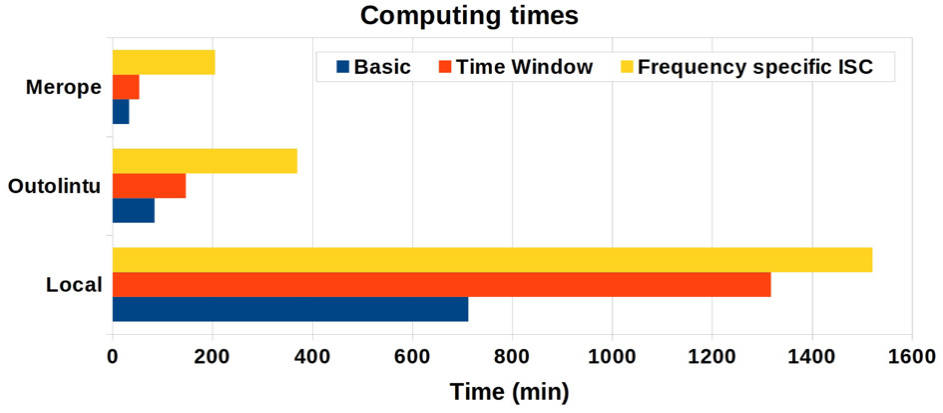
**The computing times from desktop computer and two cluster environments**. The desktop computer was equipped with Intel Core2Duo E8400 CPU 3.00 GHz and 5 GB RAM. Ten parallel processes were run on Outolintu cluster with nodes equipped with Intel Xeon X5450 CPUs 3.0 GHz. On average, 32 parallel processes were run on Merope cluster with nodes equipped with Intel Xeon X5650 CPUs 2,67 GHz.

The computing times are presented in Figure 4. On a single desktop computer, the computing time varied from 11 h 52 min to 25 h 20 min depending from the selected analysis. On the smaller Outolintu cluster, the corresponding times varied from 1 h 24 min to 6 h 10 min and, on the larger Merope cluster from 33 min to 3 h 25 min. Comparing local and distributed systems, the speed up factor was 10 with Outolintu and 24 with Merope in the first two setups. For the final setup with the frequency band analysis, the speed up factor was 4 with Outlintu and 7.5 with Merope.

The smaller speed up factors for the frequency band analysis was probably due to a higher number of hard drive interactions involved in this analysis as compared with the other tested analyses. In a cluster computing environment, a high number of hard drive interactions slows down the computations as the data is commonly located on a network drive and the speed of the data transfer in a network is usually clearly slower than the speed of data transfer via the internal bus of a desktop computer.

## 5. Discussion

Branches of neuroscience investigating brain functions in experiments mimicking real-world conditions are growing rapidly and the development of data analysis methods must address an increasing diversity of research questions. The ISC based approaches can address key questions such as how processing differs between two groups (e.g., healthy vs. nonhealthy) exposed to identical complex stimuli or between two conditions (e.g., silent vs. nonsilent video). The new analyses methods incorporated in the ISC toolbox, described in sections 2.2.4–2.2.7, are one of the first attempts to help neuroscientists to address these and other aspects of neural processing. In addition to these new features, the toolbox will be continuously updated in the future to allow even more versatile analyses. For instance, the toolbox is currently limited to analyze between-subject correlations in a voxel-wise manner and does not allow more general investigations of functional correspondence between spatially disjoint brain areas across subjects. Features to analyze ISCs between different brain areas both within- and across subjects will be incorporated in the future versions of the toolbox.

One limitation of the current analysis approach is that the used ISC measure does not capture any information about the variability of the ISCs among subjects as it is simply the average of the upper-triangular (or lower-triangular) elements of the between-subject correlation matrix computed separately for each voxel [Equation (1)]. The consequence of the averaging is that interesting features of brain processing may be missed especially in higher-order brain regions where inter-subject variability is expected to be very high. To increase the sensitivity of the existing method to localize interesting brain areas as well as to perform more fine-grained ISC based analyses, it can be highly useful to preserve and analyze the entire structure of the between-subject correlation matrices. We have already taken steps toward this direction (Kauppi et al., 2010a) and will equip the toolbox with matrix-based analysis methods in the future.

One of the key issues in ISC based analyses is how to select a suitable threshold to distinguish meaningful ISC values from spurious ones. Because of the restrictive assumptions made by standard parametric statistical procedures, such as the ordinary *t*-test, we have decided to use fully nonparametric re-sampling based methods to determine the critical thresholds to improve reliability of the analysis. Despite of the flexibility of the nonparametric methods, it is important to keep in mind that also they provide only approximations of true, underlying null re-sampling distributions. This is due to finite number of realizations drawn as well as certain assumptions required by the tests which may not be fulfilled by real fMRI time-series. However, as shown by the results, our easy and fully automated mechanism which distributes calculations across a computational cluster allows drawing huge number of realizations in a relatively short time, making the generation of accurate re-samplings distributions feasible. Moreover, we showed with a simple Monte-Carlo simulation that certain critical assumptions made by the sum ZPF test are not violated in practice. In any case, further validation and improvement of our current statistical procedures is another important topic of future research.

## 6. Conclusions

We have presented a software package, named ISC Toolbox, implemented in Matlab for computing various ISC based analyses. The computations can be launched from a GUI making the use of the toolbox easy. Many advanced techniques such as time window ISC analysis, frequency-specific ISC analysis, IPS analysis and the comparison of ISCs between different stimuli are supported by the toolbox. The analyses are coupled with nonparametric re-sampling based statistical inference methods. As these analyses are computationally intensive, the ISC Toolbox is equipped with automated cluster computing mechanisms to reduce the computation time via parallelization and a marked reduction in computation time was achieved by cluster computing. The ISC Toolbox is available in https://code.google.com/p/isc-toolbox/ under the MIT open source licence.

### Conflict of interest statement

The authors declare that the research was conducted in the absence of any commercial or financial relationships that could be construed as a potential conflict of interest.

## Footnotes

^1^By correlation coefficient, we refer to a standard Pearsons correlation coefficient.

^2^Let **s**_*i*_ denote the time course of the subject *i* that is de-meaned and normalized to unit length so that ||**s**_*i*_|| = 1 (the conclusion of this analysis does not depend on the normalization to the unit length but the analysis is simplified by that assumption). Now, *r*_*ij*_ can be written as an inner-product *r*_*ij*_ = **s**^*T*^_*i*_**s**_*j*_. Define a test statistic similarly to Lerner et al. (2011)

$$\bar{l}=\frac{1}{N}\sum_{i\;=\;1}^{N}s_{i}^{T}\left(\frac{1}{Z_{-i}(N-1)}\sum_{j\;=\;1,\;j\neq i}^{N}s_{j}\right),$$

where $Z_{-i}=||(1/(N-1))\sum_{j\;=\;1,\;j\neq i}^{N}s_{j}||$. A straight-forward computation yields

$$\begin{array}{l} \bar{l}=\frac{1}{N}\sum_{i=1}^{N}s_{i}^{T}\left(\frac{1}{(N-1)Z_{-i}}\sum_{j\;=\;1,\;j\neq i}^{N}s_{j}\right)=\frac{1}{N}\frac{1}{N-1}\sum_{i\;=\;1}^{N}\sum_{j\neq i}\frac{s_{i}^{T}s_{j}}{Z_{-i}}\\ \;=\frac{1}{N^{2}-N}\sum_{i\;=\;1}^{N}\sum_{j\neq i}\frac{r_{ij}}{Z_{-i}}=\frac{1}{\frac{N^{2}-N}{2}}\sum_{i\;=\;1}^{N}\sum_{j\;=\;2,\;j>i}^{N}\frac{r_{ij}}{Z_{-i}}, \end{array}$$

since *r*_*ij*_ = *r*_*ji*_. It can be seen that this is a weighted version of *r*, where the weights are proportional to the standard deviations of the average time courses.

^3^The analytic signal **x**_*a*_(*t*) = *x*(*t*) + *jy*(*t*) = *A*(*t*)*e*^*j*θ(*t*)^ can represent both the instantaneous amplitude envelope *A*(*t*) and phase θ(*t*) of the time-series, but only phase information $\theta (t)=\arctan\left(\frac{y(t)}{x(t)}\right)$ is used to derive our phase similarity measure.

^4^Functional MRI data from the measurements with Functional Reference Battery tasks developed by the International Consortium for Human Brain Mapping (ICBM) were used (Mazziotta et al., 2001): http://www.loni.ucla.edu/ICBM/Downloads/Downloads_FRB.shtml.
